# Mast cell activation triggered by SARS-CoV-2 causes inflammation in brain microvascular endothelial cells and microglia

**DOI:** 10.3389/fcimb.2024.1358873

**Published:** 2024-04-04

**Authors:** Meng-Li Wu, Chengzuo Xie, Xin Li, Jing Sun, Jincun Zhao, Jian-Hua Wang

**Affiliations:** ^1^Guangzhou Institutes of Biomedicine and Health, Chinese Academy of Sciences, Guangzhou, China; ^2^State Key Laboratory of Respiratory Disease, National Clinical Research Center for Respiratory Disease, Guangzhou Institute of Respiratory Health, The First Affiliated Hospital of Guangzhou Medical University, Guangzhou, Guangdong, China; ^3^University of Chinese Academy of Sciences, Beijing, China

**Keywords:** SARS-CoV-2, mast cell, neuroinflammation, tight junction protein, degranulation

## Abstract

SARS-CoV-2–induced excessive inflammation in brain leads to damage of blood–brain barrier, hypoxic-ischemic injury, and neuron degeneration. The production of inflammatory cytokines by brain microvascular endothelial cells and microglia is reported to be critically associated with the brain pathology of COVID-19 patients. However, the cellular mechanisms for SARS-CoV-2–inducing activation of brain cells and the subsequent neuroinflammation remain to be fully delineated. Our research, along with others’, has recently demonstrated that SARS-CoV-2–induced accumulation and activation of mast cells (MCs) in mouse lung could further induce inflammatory cytokines and consequent lung damages. Intracerebral MCs activation and their cross talk with other brain cells could induce neuroinflammation that play important roles in neurodegenerative diseases including virus-induced neuro-pathophysiology. In this study, we investigated the role of MC activation in SARS-CoV-2–induced neuroinflammation. We found that (1) SARS-CoV-2 infection triggered MC accumulation in the cerebrovascular region of mice; (2) spike/RBD (receptor-binding domain) protein–triggered MC activation induced inflammatory factors in human brain microvascular endothelial cells and microglia; (3) MC activation and degranulation destroyed the tight junction proteins in brain microvascular endothelial cells and induced the activation and proliferation of microglia. These findings reveal a cellular mechanism of SARS-CoV-2–induced neuroinflammation.

## 1 Introduction

SARS-CoV-2 infection causes acute respiratory distress syndrome (Jiang and Shi, 2020; Wu et al., 2020; Zhou et al., 2020). In addition to respiratory symptoms, the virus can also induce a wide range of neurological disorders such as headaches, fever, dizziness, cerebrovascular diseases, hypogeusia, neuralgia, stroke, epilepsy, impaired consciousness, encephalopathy, and psychiatric disorders, and so on (Dixon et al., 2020; Ogier et al., 2020; Wilson and Jack, 2020; Emmi et al., 2022). The persistent physical and mental fatigue is also commonly seen in the post-acute sequelae of SARS-CoV-2 (also known as “Long-COVID syndrome”). At least 20%–45% of COVID-19 convalescents have various neuropsychiatric, neurological, and neurodegenerative diseases, sleep disturbances, and cognitive deficits (Theoharides and Kempuraj, 2023). Brain autopsies of COVID-19 patients show focal subarachnoid and intraparenchymal hemorrhage, vascular congestion, and intravascular platelet aggregates (Matschke et al., 2020).

SARS-CoV-2 may enter brain by crossing neural-mucosal interface in olfactory mucosa or via the gustatory-olfactory trigeminal pathway (Jiao et al., 2021; Meinhardt et al., 2021; Beckman et al., 2022; Frank et al., 2022). The induction of excessive neuroinflammation has been reported to be a critical pathogenetic factor (Frank et al., 2022; Chaves-Filho et al., 2023; Ousseiran et al., 2023; Theoharides and Kempuraj, 2023; Li et al., 2023a, Li et al., 2023b). The neurovascular inflammation leads to the damage of blood–brain barrier (BBB), and the neuron hypoxic-ischemic injury and neuron degeneration (Bodnar et al., 2021; Lee et al., 2021; Meinhardt et al., 2021; Adesse et al., 2022; Beckman et al., 2022; Frank et al., 2022; Rutkai et al., 2022). The neuronal necrosis and cell loss, the glial cell hyperplasia, and the choroid plexus morphological changes in the microglia have been observed (Brann et al., 2020; Gomes et al., 2021; Yang et al., 2021). SARS-CoV-2 can induce microgliosis and astrogliosis and sequentially elevate the pro-inflammatory cytokines, and the damage-associated molecular pattern molecules to breakdown BBB integrity and cause neuronal degeneration (Dixon et al., 2020; Boroujeni et al., 2021; Beckman et al., 2022; Frank et al., 2022; Matschke et al., 2022; Radhakrishnan and Kandasamy, 2022; Rutkai et al., 2022; Theoharides and Kempuraj, 2023). Moreover, the infiltration of cytotoxic T lymphocytes was observed in brainstem, cerebellum, and meninges (Matschke et al., 2020). The peripheral cytotoxic T cells infiltration in the central nervous system (CNS) could further augment pro-inflammatory cytokine levels (Frank et al., 2022).

Among SARS-CoV-2 viral proteins, the spike protein has more often been reported to induce neuroinflammation and damages of brain vessels and BBB (Suprewicz et al., 2023). Spike protein can reach different brain regions, irrespective of whether there are viral replications in brain (Rhea et al., 2021; Frank et al., 2022). Spike protein exposure increases the susceptibility to angiotensin II-induced hypertension in rats, by promoting central neuroinflammation and oxidative stress (Sun et al., 2023). Spike protein may function as a pathogen-associated molecular pattern to activate microglia through the pattern recognition receptor TLR4, thus elevating neuroinflammatory cytokines (Frank et al., 2022). However, the cellular mechanisms for SARS-CoV-2–inducing neuroinflammation remain poorly understood.

Mast cells (MCs) are best known as the main effector cells in type I allergic reactions; they are increasingly recognized to act as early sentinels that sense pathogens to regulate innate and adaptive immune responses (Gilfillan and Beaven, 2011; Galli and Tsai, 2012). In brain, MCs are typically situated close to the neurovascular region on the brain side of BBB. Intracerebral MC-degranulated mediators can increase BBB permeability by breaking down the tight junctions between brain endothelial cells, and the loss of BBB integrity may further activate meningeal MCs to recruit inflammatory cells to the CNS, leading to a vicious cycle of neuroinflammation (Zhuang et al., 1996; Mattila et al., 2011; Vincent et al., 2016; Zhang et al., 2016). MC degranulation induced-neuroinflammation is associated with multiple neurological diseases (Maslinska et al., 2007; Qin et al., 2021; Bhuiyan et al., 2021a, Bhuiyan et al., 2021b).

In SARS-CoV-2 infection, the post-mortem lung biopsies of COVID-19 patients show a massive increase in the density of perivascular and septal MCs (Motta Junior et al., 2020; Budnevsky et al., 2022; Schaller et al., 2022). Our research, along with others’, has recently reported that SARS-CoV-2–induced massive MC accumulation and rapid degranulation caused lung inflammation and damage both in mice and nonhuman primate models (Wu et al., 2021, 2022; Tan et al., 2023). The severity of SARS-CoV-2 infection is associated with high number of alveolar MCs and their degranulation (Krysko et al., 2022). The activated MCs have been proposed to further stimulate microglia and other brain cells to release inflammatory and neurotoxic mediators that cause neuroinflammation and exacerbate neuro-COVID disease severity (Theoharides and Kempuraj, 2023). To investigate the potential role of MCs in SARS-CoV-2–induced neuroinflammation, we examined the effects of spike protein-triggered MC degranulation on human brain microvascular endothelial cells and microglia cells.

## 2 Materials and methods

### 2.1 Cells and virus

Human brain microvascular endothelial cells hCMEC/D3 (purchased from Meisen CTCC, Zhejiang, China) and microglial cells HCM3 (purchased from Meisen CTCC, Zhejiang, China) were cultured in DMEM medium (Gibco, Invitrogen, California, USA) containing 10% fetal bovine serum (FBS) (Gibco), 100 U/mL penicillin and 100 μg/mL streptomycin. Human mast cells LAD2 were cultured in RPMI 1640 medium (Gibco), containing 10% fatal FBS with 100 U/mL penicillin and 100 μg/mL streptomycin. For degranulation, LAD2 cells (purchased from Huzhen Company, Shanghai, China) were grown in StemPro-34 medium (Gibco) supplemented with 100 μg/ml stem cell factor (Novoprotein, Suzhou, China), 100 μg/ml IL-6 (Novoprotein), nutrient supplement (Gibco), 100 U/ml penicillin (Invitrogen, California, USA), 100 µg/ml of streptomycin (Invitrogen), and 2 mM L-Glutamine (Gibco).

HIV-NL4-3/spike pseudotyped virus was generated by Lipo 2000 transfection reagent (Invitrogen)-mediated co-transfection of HEK293T cells, with the spike-expressing plasmid pcDNA3.1-2019-nCoV-S-IRES (strain 2019-nCoV WIV04) and pNL4-3. Luc. ΔR ΔE. These two plasmids and HEK293T cells are provided by Dr. Lu Lu (Fudan University, Shanghai, China). Harvested HIV-NL4-3/spike pseudovirus were used to infect hCMEC/D3 and HMC3 cells for 48h, viral infection was measured by detecting luciferase activity (Promega, Madison, WI, USA).

### 2.2 Flow cytometry, cell proliferation, and Western blotting

For detecting the expression of HLA-DR and ACE2, cells were incubated with anti-human HLA-DR allophycocyanin (APC) (Tonbo Biosciences, San Diego, USA, 20-9952) or anti-human ACE2-phycoerythrin (PE) (Bioss, Beijing, China, bs-1004R) at 4°C for 30 min and detected with flow cytometry (BD Accuri C6). For detecting the expression of ZO-1, JAM2, and OCLN in hCMEC/D3 cells, cells (1 × 10^5^) were treated with spike-RBD proteins (2 μg/mL) derived from the wild type of SARS-CoV-2 (GenScript, Nanjing, China, Z03483), or co-cultured with LAD2 cells (1 × 10^5^) in the presence or absence of spike-RBD proteins (2 μg/mL), or stimulated with RBD-treated LAD2 cell culture supernatants (300 μL), or histamine (5 μg/mL), tryptase (5 μg/mL, or chymase (5 μg/mL) for 48h. In some samples, loratadine (Lor.). (2.5 μg/mL, Selleck, Houston, Texas, USA), ebastine (Eba.) (1.5 μg/mL, Selleck), or ketotifen fumarate (Ket.) (4 μg/mL, Yuanye Biology, China, S46226), was used to prior-treat cells for 2h before the next treatment. Cells were harvested and blocked with 5% BSA for 1h at room temperature and incubated with primary antibody for 2h. Primary antibodies against ZO-1 (Invitrogen, 402200), OCLN (Invitrogen, OC-3F10), and JAM-2 (Abcam, Cambridge, UK, EPR2489) were used. Cells were washed with FACS buffer and incubated with Alexa Flour 488–labeled goat anti-rabbit (Invitrogen, A11034) for 30 min and were analyzed with flow cytometry (BD Accuri C6).

HMC3 cells were pre-labeled with CFSE (carboxyfluorescein succinimidyl ester) (Invitrogen) for 10 min at 37°C; the reaction was terminated with 40% FBS and washed three times with phosphate buffered saline (PBS), then HMC3 cells were co-cultured with LAD2/RBD or treated with histamine (5 μg/mL), tryptase (5 μg/mL), or chymase (5 μg/mL) for 24h at 37°C. Cells were analyzed with flow cytometry (BD Accuri C6).

For Western blotting, cells were lysed for 30 min at 4°C in lysis buffer (Beyotime, Shanghai, China). After centrifugation for 10 min at 12,000 g, the supernatant was boiled in reducing SDS sample loading buffer and analyzed by SDS-PAGE. The anti-ZO-1 antibody (Invitrogen, 402200), anti-OCLN antibody (Invitrogen, OC-3F10), anti-JAM-2 antibody (Abcam, EPR2489), and anti-GAPDH antibody (Abcam, ab82633), and the horseradish peroxidase–conjugated secondary antibody were used in Western blotting.

### 2.3 Histology

Specific pathogen-free 6-week-old female wild-type BALB/c mice were infected intranasally with the 501Y.V2 SARS-CoV-2 variant (B.1.351, GISAID: EPI_ISL_2423556) at a 50% cell culture infective dose (CCID_50_) of 7 × 10(4). The 501Y.V2 strain was provided by Guangzhou Medical University, Guangdong, China. Five mice were in each group and three mice were in control group. The brains were collected at 3 days post-infection (dpi) for pathological, virological, and immunological analyses. The brains of mice were harvested and fixed in zinc formalin. For routine histology, tissue sections (~4 μm each) were stained with hematoxylin and eosin (H&E) or toluidine blue (T. blue) (Sigma-Aldrich, USA). In brief, the tissue sections were stained for 1h at room temperature, and then washed in distilled water three times, anhydrous ethanol two times, and covered with a coverslip. The sections were analyzed using a Pannoramic MIDI slice scanning apparatus (3DHISTECH, USA). The fixed tissue sections were stained at the Pathology Center of the GIBH, CAS.

### 2.4 Real-time PCR

Total cellular RNAs were extracted by using TRIzol Reagent (Invitrogen) and then reverse transcribed into cDNA with synthesis Kit (TOYOBO, Shanghai, China, FSQ-301), according to the manufacturer’s instructions. Real-time polymerase chain reaction (PCR) was carried out by using the SYBR qPCR Mix (Genestar, Beijing, China, A33-101) with the following thermal cycling conditions: initial denaturation at 95°C for 2 min, amplification with 40 cycles of denaturation at 95°C for 15 s, primer annealing at 60°C for 15 s, and extension at 72°C for 30s. The data were analyzed by SYBR green-based semi-quantification and normalized with GAPDH. Real-time PCR was performed on the Bio-Rad CFX96 real-time PCR system. The primers and probes for (RT-) PCR were listed in **Supplementary Table S1**.

### 2.5 RNA-seq and data analysis

hCMEC/D3 cells were treated with spike RBD–treated LAD2 cell culture supernatants or LAD2 normal culture supernatants for 24h. Total RNAs were extracted using Trizol (Invitrogen) according to the manufacturer’s protocol, and ribosomal RNA removed using QIAseq FastSelect-rRNA HMR Kits (QIAGEN, Germany). Fragmented RNAs (average length approximately 200 bp) were subjected to first strand and second strand cDNA synthesis, followed by adaptor ligation and enrichment with a low-cycle according to the instructions of NEBNext UltraTM RNA Library Prep Kit for Illumina (NEB, USA). The purified library products were evaluated using the Agilent 2200 TapeStation and Qubit2.0 (Life Technologies, USA). The libraries were paired-end sequenced (PE150, sequencing reads were 150 bp) at Genewiz (Jiangsu, China) using Illumina HiSeq 3000 platform. The data analysis was according to the reference (Wu et al., 2021).

### 2.6 Statistical analysis

Graphpad Prism 8.0 was used for statistical analysis. Student’s unpaired two-tailed *t*-test was performed to analyze significant difference. Significance levels are indicated as *p < 0.05, **p < 0.01, ***p < 0.001.

## 3 Results

### 3.1 SARS-CoV-2 infection induces MC accumulation around cerebrovascular region in mice

**Figure 1:**
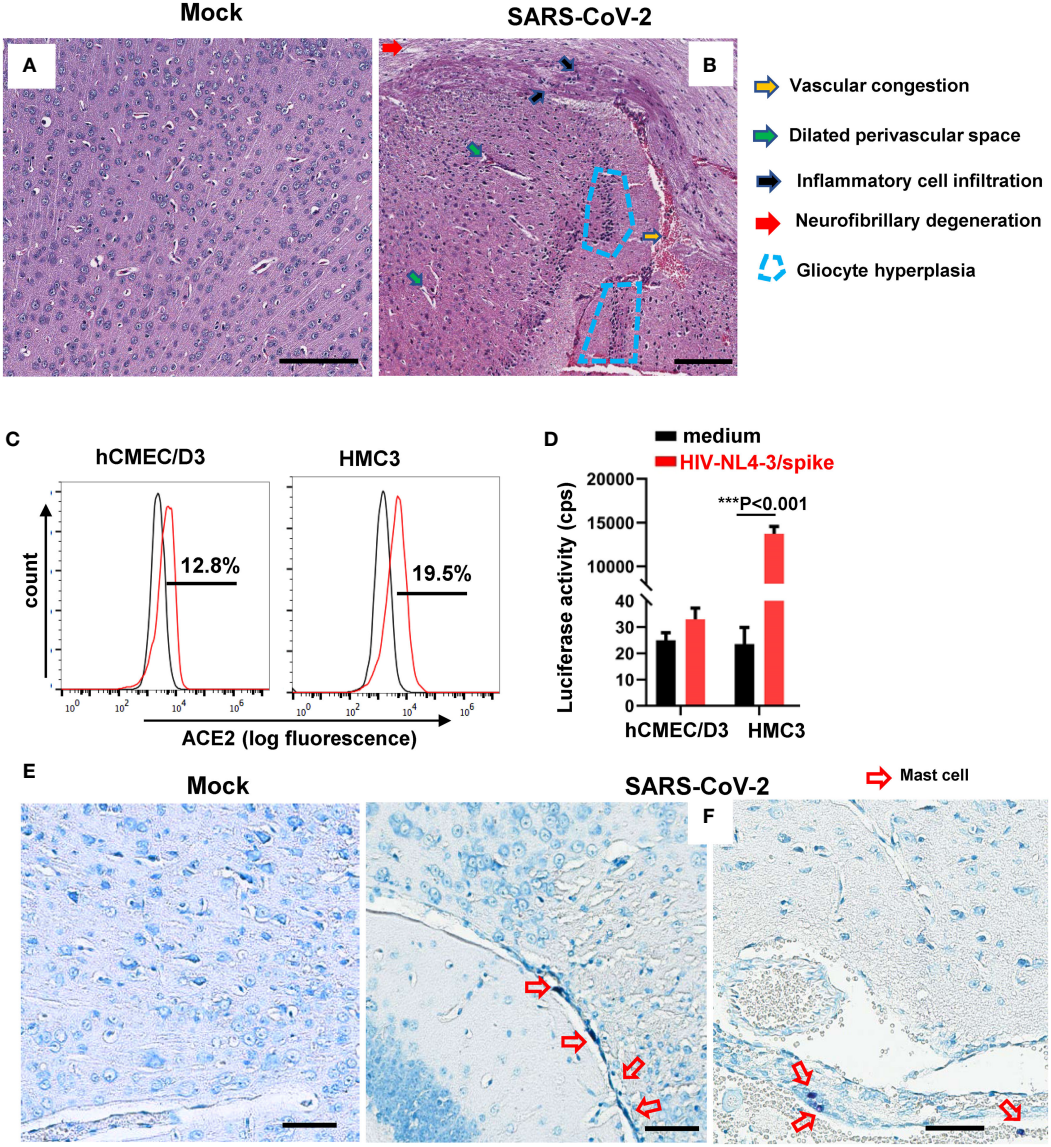
SARS-CoV-2 infection induces MC accumulation around cerebrovascular region in mice. BALB/c mice were infected intranasally with SARS-CoV-2 501Y.V2 strain at the dose of 7 × 10^4^ CCID_50_. The brains were collected at 3 dpi. Brain injury was observed by H.E. staining of brain sections **(A, B)** and T. blue staining was used to observe MCs **(E, F)**. Scale bar: 50 µm. **(C)** ACE2 expression in hCMEC/D3 and HMC3 detected by flow cytometry with immunostaining with specific antibody. **(D)** Viral infection. hCMEC/D3 and HMC3 cells were infected with SARS-CoV-2 spike-pseudotyped lentivirus (HIV-NL4-3/spike) (5 ng p24^gag^) for 48h, and viral infection was determined by measuring the luciferase activity. One representative result from three independent repeats is shown. Data are presented as *M* ± *SD*. ***p < 0.001 is considered significant differences.

The autopsies of COVID-19 patients reveal brain tissue lesions, including hemorrhage, perivascular inflammatory cell infiltration, intravascular platelet aggregates, vascular congestion, and so forth (Matschke et al., 2020). We recapitulated these observations in experimentally SARS-CoV-2–infected mice. BALB/c mice were intranasally infected with the SARS-CoV-2 (501Y.V2, 7 × 10^4^ CCID_50_), then euthanized at 3 days post-infection (dpi) to harvest brain tissues for histopathology analysis. Mock infection was performed using the same amount of PBS (**Figure 1A**). Compared to the mock-infection control, the H&E staining of brain sections showed the vascular congestion, dilated perivascular space, inflammation cell infiltration, neurofibrillary degeneration, and gliocyte hyperplasia in SARS-CoV-2–infected mice (**Figure 1B**).

Because neurons, astrocytes, oligodendrocytes, and glial cells can be infected with SARS-CoV-2 through viral binding with ACE2 and TMPRSS2 (McQuaid et al., 2021; Pacheco-Herrero et al., 2021), we next investigated the susceptibility of microvascular endothelial cells and microglial cells to SARS-CoV-2 infection. Both human brain microvascular endothelial cells hCMEC/D3 and the microglial cells HCM3 expressed ACE2 receptor (**Figure 1C**). The HMC3 cells, but not hCMEC/D3 cells, showed susceptibility to infection with SARS-CoV-2 spike-pseudotyped lentivirus (NL4-3/spike) (**Figure 1D**).

To investigate the potential role of MCs in SARS-CoV-2 infection–caused brain lesion, the brain tissue sections of SARS-CoV-2–infected mice were stained with T. blue to indicate MCs (Wu et al., 2021, 2022). Compared to mock infection (**Figure 1E**), the accumulation of MCs around cerebrovascular region was observed (**Figure 1F**), demonstrating that SARS-CoV-2 infection induced MC accumulation around cerebrovascular region.

### 3.2 MC degranulation induces inflammatory factors in microglia and brain microvascular endothelial cells, leading to microglial activation and proliferation

**Figure 2:**
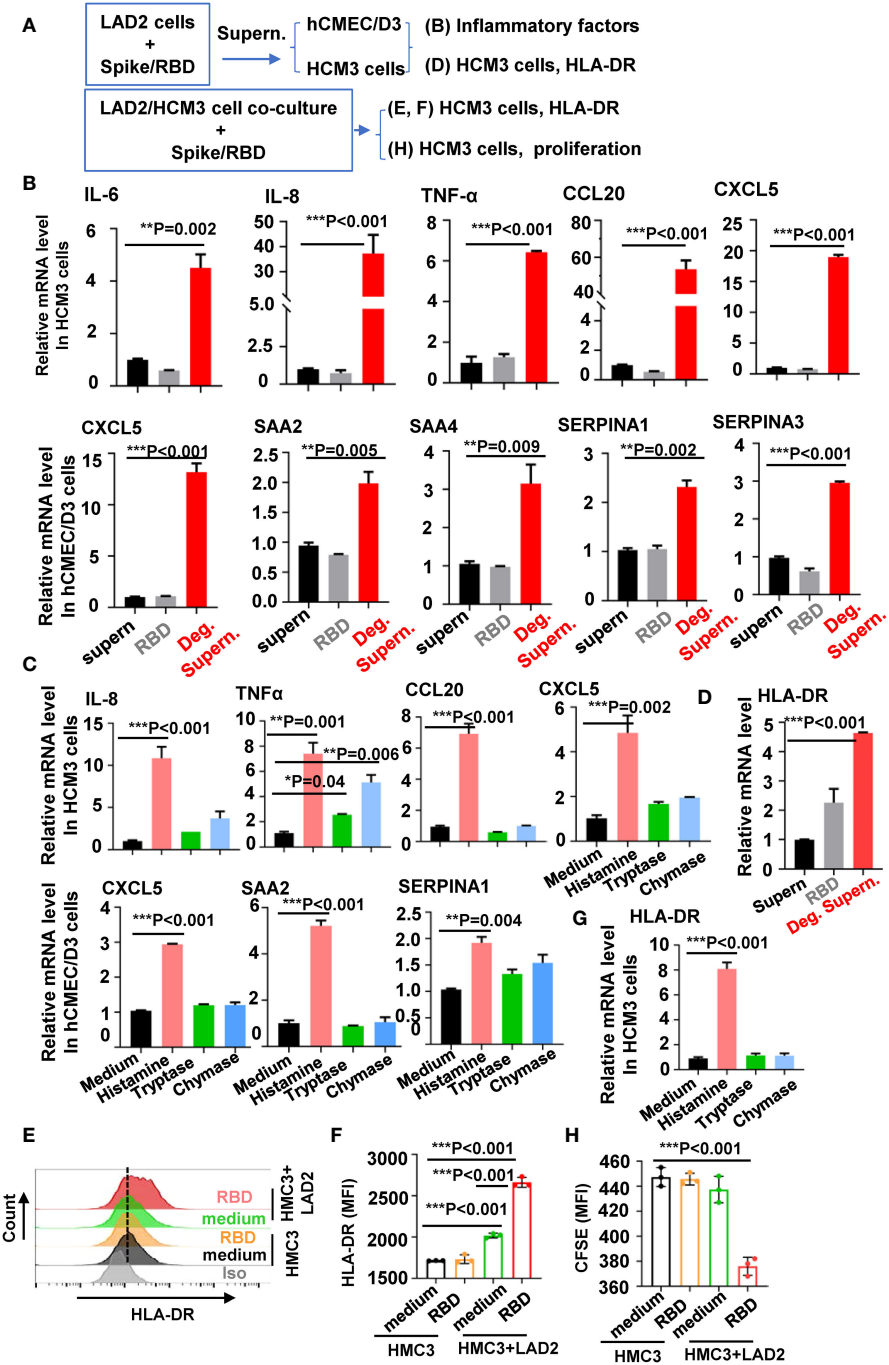
MC degranulation induces the inflammatory factors and proliferation of brain cells. **(A)** Flow chat for experiment design. The hCMEC/D3 and HMC3 cells (2 × 10^5^) were treated with RBD-stimulated LAD2 cell Deg. Supern. (300 µL), LAD2 normal cell culture supernatant (supern.), or with RBD (2 µg/mL) for 24h **(B)**, or cells were treated with histamine (5 µg/mL), tryptase (5 µg/mL), and chymase (5 µg/mL) for 24h **(C)**. Cellular mRNA levels of cytokines were quantified with q(RT)-PCR and normalized to *gapdh* mRNA. **(D)** HMC3 cells were treated with RBD (2 µg/mL), RBD-stimulated LAD2 cell Deg. Supern. (300 µL), or LAD2 normal cell culture supernatant (supern.), for 24h, the cellular mRNA level of HLA-DR was quantified with q(RT)-PCR. HMC3 cells alone or were co-cultured with LAD2 cells in presence or absence of RBD protein (2 µg/mL) at 37°C for 48h, the adherent HMC3 cells were harvested to detect HLA-DR expression with flow cytometry **(E, F)**. **(G)** HMC3 cells were treated with histamine (5 µg/mL), tryptase (5 µg/mL) and chymase (5 µg/mL) for 48h, HLA-DR expression was detected with quantified with q(RT)-PCR. **(H)** Cell proliferation. HMC3 cells were pre-labeled with CFSE and then co-cultured with LAD2 cells in presence or absence of RBD protein for 24h at 37°C, the adherent HMC3 cells was harvested and analyzed with flow cytometry. Data are presented as *M* ± *SD*. *p < 0.05, **p < 0.01, and ***p < 0.001 are considered as significant differences.

MC-degranulated mediators could further activate microglia and other brain cells to release inflammatory and neurotoxic mediators that could cause neuroinflammation and exacerbate neuro-COVID disease severity (Theoharides and Kempuraj, 2023). We thus investigated the role of MC degranulation in inducing inflammatory factors in human microglial cells and brain microvascular endothelial cells (**Figure 2A**). The MC cell line LAD2 can be triggered by SARS-CoV-2 spike/RBD protein for rapid degranulation (Wu et al., 2021, 2022). The spike/RBD protein-triggered MC degranulation led to release of factors into cell culture supernatants (Deg. Supern.), which were used to treat hCMEC/D3 and HCM3 cells for 24h. Compared to the treatment with normal cell culture supernatant of LAD2 (supern.), the treatment with MC Deg. Supern. significantly stimulated the expression of inflammatory factor IL-6, IL-8, TNF-α, CCL20, and CXCL5 in HCM3 cells, and elevated the expression of CXCL5, SAA2, SAA4, SERPINA1, and SERPINA3 in hCMEC/D3 (**Figure 2B**). Whereas the direct stimulation with SARS-CoV-2 spike/RBD protein for 24h did not induce the expression of these inflammatory factor in either cell line (**Figure 2B**).

MC granules include multiple biologic mediators including histamine, serotonin, heparin, cytokine/chemokines, and enzymes such as chymase and tryptase (Elieh Ali Komi et al., 2020). We have previously detected the rapid release of histamine, chymase and tryptase in SARS-CoV-2 spike/RBD protein-treated LAD2 cells (Wu et al., 2021, 2022). To profile the mediators-induced expression of inflammatory factors, cells were treated with histamine, tryptase or chymase. The treatment with histamine, but not with tryptase or chymase, induced the significant expression of inflammatory factors (**Figure 2C**).

Microglial activation and proliferation are the common pathology found in the brain autopsy samples of COVID-19 individuals (Matschke et al., 2020; Theoharides and Kempuraj, 2023). We investigated the stimulation of MC degranulation on microglial activation and proliferation. Microglial activation can be determined by the elevated immunostaining of markers HLA-DR, IBA1 (ionized calcium-binding adaptor molecule 1), and CD68 (Al-Dalahmah et al., 2020; Kantonen et al., 2020; Matschke et al., 2020; Rutkai et al., 2022). The treatment of HCM3 cells with MC Deg. Supern. significantly elevated HLA-DR expression (**Figure 2D**). To confirm this, the LAD2/HMC3 cell co-culture was stimulated with spike/RBD protein, and elevated HLA-DR expression was observed (**Figures 2E, F**). In comparison, the direct treatment with spike/RBD protein did not induce HLA-DR expression in HMC3 cells (**Figures 2D–F**). The released histamine might account for the induction of HLA-DR, as the treatment with histamine, but not with tryptase or chymase, elevated HLA-DR expression (**Figure 2G**). Spike/RBD protein-triggered degranulation during the LAD2/HMC3 cell co-culture induced HMC3 proliferation, as detected with CFSE staining and FACS analysis (**Figure 2H**). Taken together, these results demonstrate that MC degranulation induces inflammatory factors in microglia and brain microvascular endothelial cells, and induces microglial activation and proliferation.

### 3.3 Transcriptome analysis reveals MC degranulation inducing inflammatory factors in human brain microvascular endothelial cells

**Figure 3:**
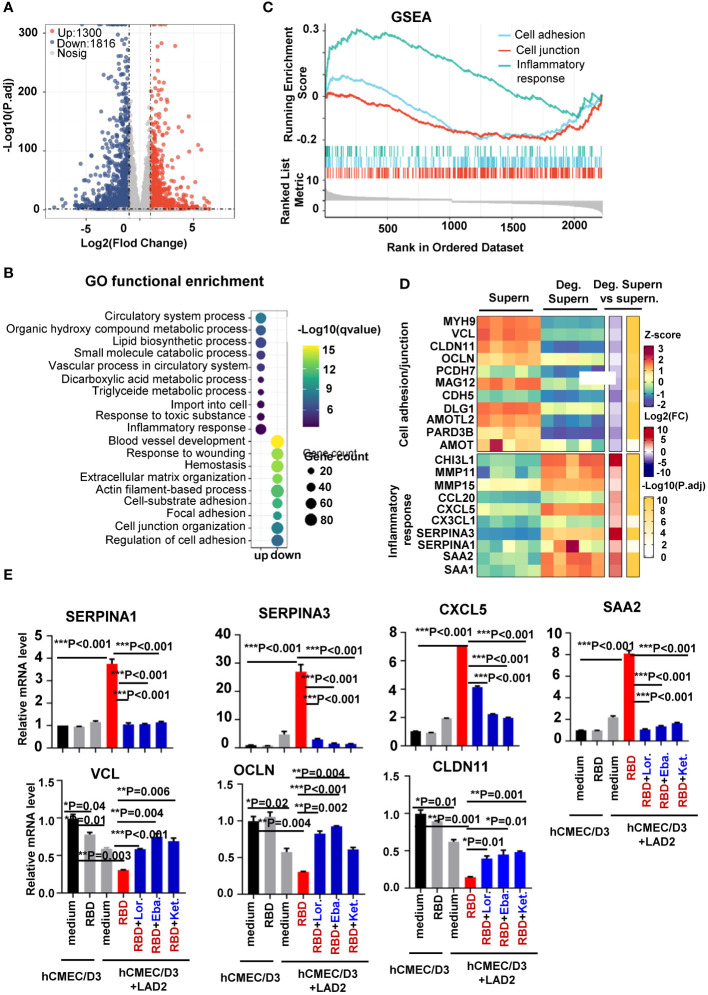
Transcriptome analysis of hCMEC/D3 cells treated with LAD2 cell degranulation supernatants. Volcano plot of DEGs comparing the treatment with spike/RBD-triggered LAD2 cell Deg. Supern. versus LAD2 normal culture supernatants (supern.). Transcriptomes were analyzed using standard protocols and data from five independent repeats were summarized. The symbols of upregulated or downregulated genes are shown **(A)**. GSEA showing the distribution of gene sets that related to inflammatory response and the enrichment scores based on DEGs **(B)**. GO functional enrichment analysis of DEGs. The color bar indicates the minus logarithm of q values, and bubble size indicates the absolute gene counts enriched in a GO term **(C)**. Heatmaps showing relative expression level (left panel), fold change (middle panel), and adjusted *p*-values (right panel) for cell adhesion/junction and inflammatory response-related genes **(D)**. **(E)** Prior treatment with antihistamines blocked expression of inflammatory factors. hCMEC/D3 cells were prior treated with Lor. (2.5 µg/mL), Eba. (1.5 µg/mL), or Ket. (4 µg/mL), for 2h before coculture with LAD2 cells. The cellular mRNA levels of cytokines were quantified with real time q(RT)-PCR and normalized to gapdh mRNA. Result is summarized from three independent repeats. Data are presented as *M* ± *SD*. *p < 0.05, **p < 0.01, and ***p < 0.001 are considered as significant differences.

To globally monitor MC degranulation-induced alteration of cellular signaling in human brain microvascular endothelial cells, the transcriptome was analyzed. hCMEC/D3 cells were treated with spike/RBD protein-triggered LAD2 cell Deg. Supern. for 24h. Transcriptomes were analyzed using standard protocols. Compared with the treatment with normal LAD2 cell culture supernatant (Supern.), the treatment with Deg. Supern. upregulated 1,300 and downregulated 1,816 genes (**Figure 3A**). Gene ontology functional enrichment analysis of differentially expressed genes (DEGs) showed obvious upregulation of gene sets involved in inflammation response and downregulation of gene sets involved in cell junction organization and regulation of cell adhesion (**Figure 3B**). The gene set enrichment analysis (GSEA) linked the upregulated genes to the regulation of inflammation response and the downregulated genes to the regulation of cell adhesion and cell junction (**Figure 3C**). The core DEGs involved in the regulation of cell adhesion/junction and inflammation were catalogized. The upregulated core DEGs linked to inflammation response mainly included *CCL20, CXCL5, CX3CL1, SERPINA3, SERPINA1, SAA2* and *SAA1*, and the downregulated core DEGs linked to cell adhesion/junction mainly included *MYH9*, *VCL*, *CLDN11*, and *OCLN* (**Figure 3D**).

The core DEGs were confirmed with real-time PCR. The addition of SARS-CoV-2 spike/RBD protein in the co-culture of hCMEC/D3-LAD2 cells significantly elevated the expression of *SERPINA3*, *SERPINA1*, *CXCL5*, and *SAA2* and downregulated the expression of *VCL*, *OCLN*, and *CLDN11* in hCMEC/D3 cells (**Figure 3E**). The direct stimulation of hCMEC/D3 with spike/RBD protein did not alter these expressions (**Figure 3E**).

We have above demonstrated that the released histamine was a key factor for triggering the production of inflammatory cytokines. To verify this, the antihistamines Lor., Eba., and Ket. were used in the next set of experiments. hCMEC/D3-LAD2 cells were treated with these antihistamines prior to co-culture, and this led to reduced expression of inflammatory factors in hCMEC/D3-LAD2 cells during subsequent co-culture (**Figure 3E**). Taken together, this transcriptome analysis reveals the alteration of cellular signaling triggered by MC degranulation in human brain microvascular endothelial cells.

### 3.4 MC degranulation disrupts the tight junction proteins in brain microvascular endothelial cells

**Figure 4:**
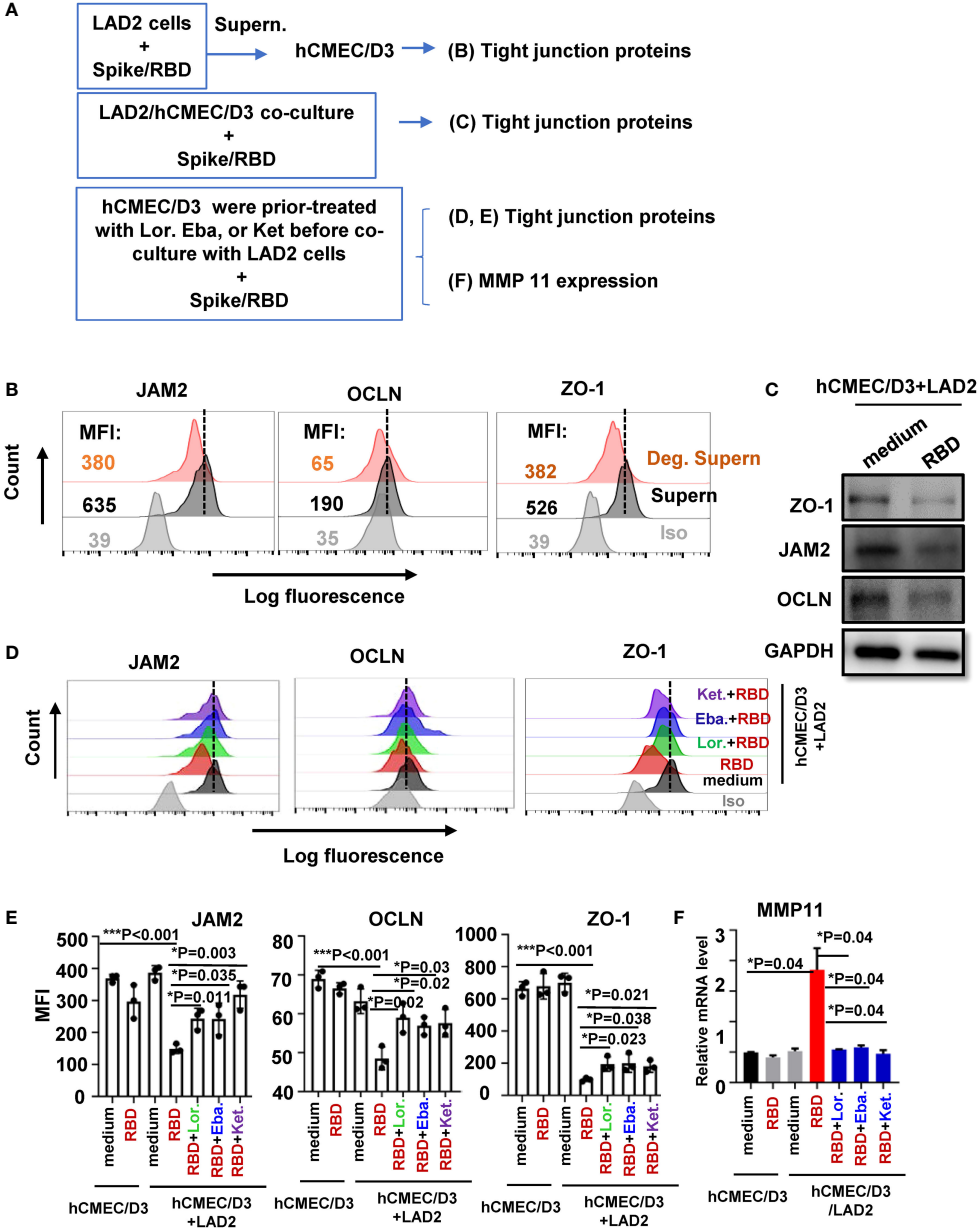
MC degranulation disrupts tight junction protein in hCMEC/D3 cells. **(A)** Flowchart for experiment design. hCMEC/D3 cells (2 × 10^5^) were treated with RBD-stimulated LAD2 cell Deg. Supern. (300 μL) or LAD2 normal culture supernatants (supern.) for 48h **(B)**, or were co-cultured with LAD2 cells in presence or absence of RBD protein (2 μg/mL) at 37°C for 48h **(C, D)** and in **(D, E)** hCMEC/D3 cells were prior treated with Lor. (2.5 μg/mL), Eba. (1.5 μg/mL), or Ket. (4 μg/mL) for 2h before coculture with LAD2 cells. Tight junction proteins expressions were detected with flow cytometry **(B, D, E)** and Western blotting **(C)**. The mean fluorescence intensity (MFI) was calculated **(E)**. **(F)** The treated hCMEC/D3 cells as above were harvested to detecting MMP11 expression with q(RT)-PCR. Result is summarized from three independent repeats. Data are presented as *M* ± *SD*. *p < 0.05 and ***p < 0.001 are considered as significant differences.

We have recently demonstrated that spike/RBD protein-induced MC degranulation disrupted barrier integrity of alveolar epithelial and pulmonary microvascular endothelial cells (Wu et al., 2021; Wu et al., 2022), and MC accumulation can be found around cerebrovascular region in SARS-CoV-2–infected mice. In this study, we further examined the effect of MC degranulation on the integrity of human brain microvascular endothelial cells, by detecting the expression of tight junction proteins (**Figure 4A**). Spike/RBD protein-treated LAD2 cell Deg. Supern. was used to treat hCMEC/D3 cells for 48h, the expression of JAM2, OCLN, and ZO-1 were detected. The treatment with Deg. Supern. reduced these proteins, as detected with flow cytometry (**Figure 4B**). To confirm this, the LAD2-hCMEC/D3 cell co-culture was used. The addition of spike/RBD protein to the co-culture reduced the expression of these proteins in hCMEC/D3 cells (**Figures 4C–E**). As expected, the direct treatment of hCMEC/D3 cells with spike/RBD protein for 48h did not downregulate the expression of these tight junction proteins (**Figure 4E**). Moreover, the prior treatment of hCMEC/D3 cells with antihistamines Lor., Eba., and Ket. rescued the reduction of tight junction proteins (**Figures 4D, E**).

We have previously demonstrated that the matrix metallopeptidase could be induced by MC degranulation in alveolar epithelial cells to disrupt the barrier integrity (Wu et al., 2021). The addition of spike/RBD protein to the co-culture of LAD2/hCMEC/D3 induced the MMP11 expression in hCMEC/D3 cells, and prior treatment of hCMEC/D3 cells with antihistamines reduced MMP11 induction (**Figure 4F**). Taken together, these data demonstrate that MC degranulation disrupts the tight junction proteins in brain microvascular endothelial cells.

## 4 Discussion

**Figure 5:**
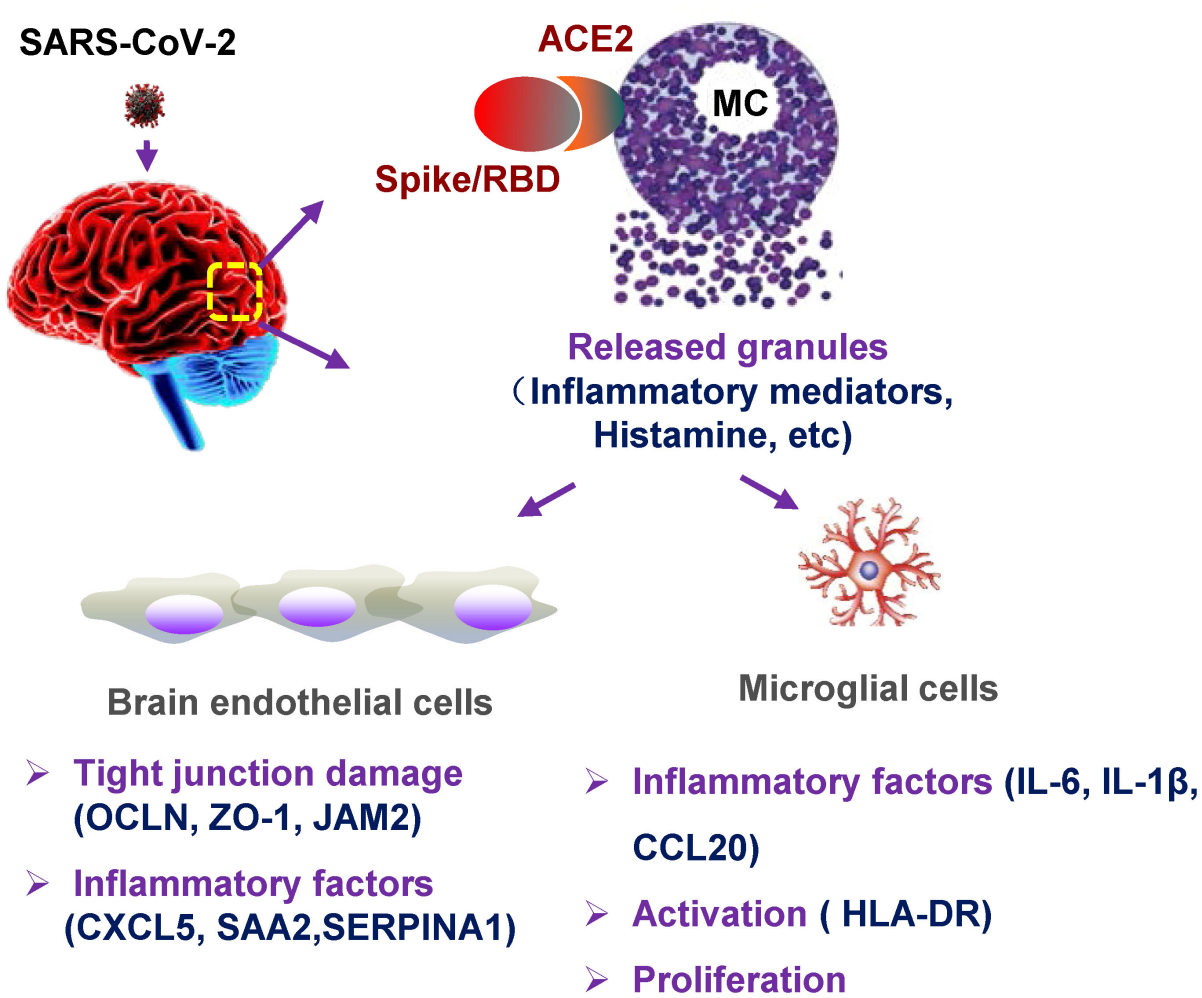
Graphical abstract. MC degranulation causes inflammation in brain microvascular endothelial cells and microglia. The treatment with SARS-CoV-2 spike protein triggers MC degranulation, and the degranulated component such as histamine induces the inflammatory factors CXCL5, SAA2, SERPINA1 in human brain microvascular endothelial cells, and reduced the tight junction proteins of OCLN, ZO-1, and JAM2; MC degranulation also increases the expression of inflammatory factors IL-6, IL-1β, and CCL20 in microglial cells, and induces the activation and proliferation of microglia.

The cellular mechanism for SARS-CoV-2–inducing neuroinflammation remains unclear. Intracerebral MCs are situated close to the neurovascular region, and MC-degranulated mediators may disrupt BBB integrity, hence, promote the infiltration of inflammatory cells, and further cause brain edema and hemorrhage (Qin et al., 2021; Bhuiyan et al., 2021a, Bhuiyan et al., 2021b). In this study, we focused on investigating the role of MCs in SARS-CoV-2–induced neuroinflammation. We found that SARS-CoV-2 infection triggered MC accumulation in the cerebrovascular region of mice, and MC degranulation induced inflammatory factors in human brain microvascular endothelial cells and microglia, together, these reduced the expression of tight junction proteins (**Figure 5**). The finding of intracerebral MC degranulation suggests a cellular mechanism to understand SARS-CoV-2–induced neuroinflammation.

Other than brain, the rapidly induced MC degranulation in various tissues, including lung, trachea, heart, and intestines, could offer an explanation of multi-focal SARS-CoV-2–induced inflammation and damages (our submitted data). Our research, along with others’, has recently reported that SARS-CoV-2–induced MC massive accumulation and rapid degranulation caused lung inflammation and injury in mice and nonhuman primates (Wu et al., 2021, 2022; Tan et al., 2023). The severity of SARS-CoV-2 infection is associated with high number of alveolar MCs and their degranulation (Krysko et al., 2022). Moreover, the sera of post-acute sequelae of COVID-19 patients display a distinct profile of elevated inflammatory cytokines and MC-released proteases, suggesting the association of MC-induced systemic inflammation with long-COVID (Wechsler et al., 2022). Overall, the observation of SARS-CoV-2–induced rapid and long-lasting MC activation and degranulation in various tissues provides a new clue for revealing virus-induced tissue inflammation and injury.

The induced MC degranulation has been implicated in various types of virus-induced brain pathophysiology, and it may be a common mechanism. MCs response to viruses could promote detrimental inflammation and disrupt a variety of barrier system to allow viruses to enter tissues where they would otherwise unable to do. The degranulated chymase triggered by Japanese encephalitis virus (JEV) can enhance brain vascular leakage, increase JEV infection in CNS, and reduce the survival of infected mice (Hsieh et al., 2019). MC accumulation, activation, and allergic inflammation have been found in the brains, lungs, and skeletal muscle of EV71-infected mice, accompanying with the elevated level of IL-4, IL-5, IL-13, and TNF-α in brain (Jin et al., 2018). Dengue virus induces MC to secrete vasoactive products, including TNF, chymase, histamine, serotonin, and VEGF, which can act directly on vascular endothelium, increasing permeability and inducing vascular leakage (St John et al., 2013; Wan et al., 2018).

MCs can interact with various immune cells through release of soluble factors or direct contact. Microglial activation has been found in the SARS-CoV-2–infected non-human primates and the post-mortem of COVID-19 patients (Dixon et al., 2020; Boroujeni et al., 2021; Beckman et al., 2022; Matschke et al., 2022; Radhakrishnan and Kandasamy, 2022; Rutkai et al., 2022; Theoharides and Kempuraj, 2023). The activated MCs have been proposed to activate microglia to release inflammatory and neurotoxic mediators that cause neuroinflammation and exacerbate neuro-COVID disease severity (Theoharides and Kempuraj, 2023). This study proves that MC degranulation activates microglia cells, promotes cell proliferation, and induces inflammatory factors.

The reduction of tight junction protein in brain microvascular endothelial cells induced by MC degranulation may disrupt BBB integrity and thus provide a feasible condition for the infiltration of inflammatory cells. We have observed that MC degranulation upregulated the expression of CXCL5, CCL20, SAA2, SAA1, SERPINA1, and SERPINA3 in brain microvascular endothelial cells. The inflammatory factors SAA2, SAA1, SERPINA1, and SERPINA3 are significantly upregulated proteins in the sera of the severe COVID-19 patients (Shen et al., 2020; Taz et al., 2021; Cui et al., 2022).

The limitation of this study is that the finding mainly depends on the infected mouse model and cell lines. The post-mortem lung biopsies of COVID-19 patients show a massive increase in the density of perivascular and septal MCs (Motta Junior et al., 2020; Budnevsky et al., 2022; Schaller et al., 2022). Hopefully, our study would provide a clue to investigate MC role in physiological condition.

Taken together, in this study, we found that (1) SARS-CoV-2 infection triggered MC accumulation in the cerebrovascular region of mice, (2) spike/RBD protein-triggered MC degranulation induced the expression of inflammatory factors in human brain microvascular endothelial cells and microglia, and (3) MC degranulation reduced the tight junction proteins in brain microvascular endothelial cells and the activation and proliferation of microglia. These findings reveal a cellular mechanism for SARS-CoV-2–induced neuroinflammation.

## Data availability statement

The datasets presented in this study can be found in online repositories. The names of the repository/repositories and accession number(s) can be found below: https://www.ncbi.nlm.nih.gov/bioproject/PRJNA896725, PRJNA896725.

## Ethics statement

Ethical approval was not required for the studies on humans in accordance with the local legislation and institutional requirements because only commercially available established cell lines were used. The animal study was approved by the Institutional Animal Care and Use Committee of the Guangzhou Institutes of Biomedicine (GIBH), Chinese Academy of Sciences (CAS), Guangzhou, China. The study was conducted in accordance with the local legislation and institutional requirements.

## Author contributions

M-LW: Data curation, Formal analysis, Investigation, Methodology, Software, Validation, Visualization, Writing – original draft. CX: Data curation, Formal analysis, Investigation, Methodology, Software, Validation, Visualization, Writing – original draft. XL: Investigation, Methodology, Writing – original draft. JS: Methodology, Resources, Visualization, Writing – original draft. JZ: Methodology, Resources, Writing – original draft. J-HW: Conceptualization, Formal analysis, Funding acquisition, Investigation, Project administration, Resources, Supervision, Writing – original draft, Writing – review & editing.

## Supplementary material

Supplementary Table 1 The primers for real-time PCR. The primers used for real-time PCR were listed.
