# Supplementary figures and images for: Mast cell activation triggered by SARS-CoV-2 causes inflammation in brain microvascular endothelial cells and microglia
Source: Front Cell Infect Microbiol. 2024 Apr 4;14:1358873. doi: 10.3389/fcimb.2024.1358873 (PMC11024283; doi:10.3389/fcimb.2024.1358873)

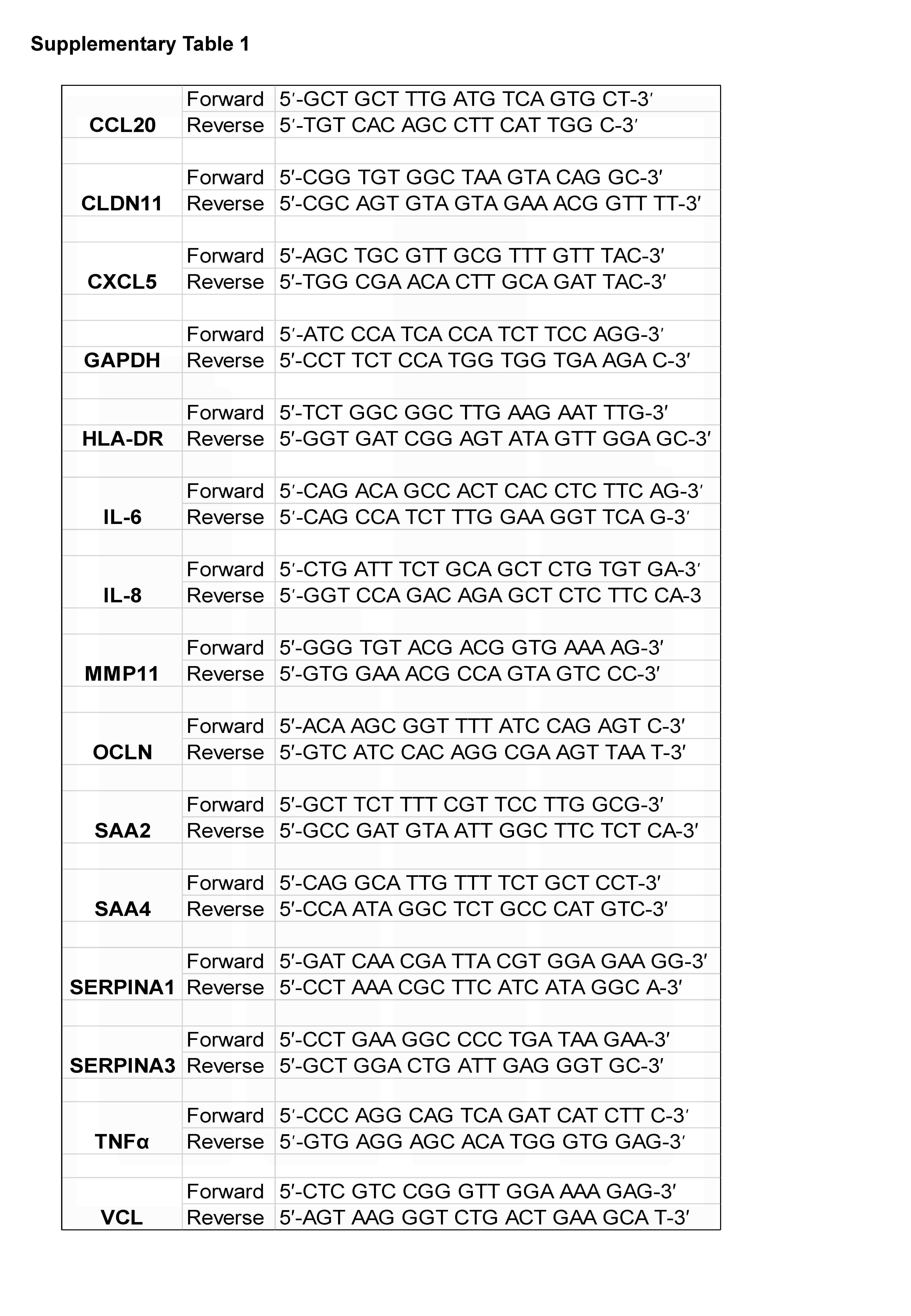

Supplement: Supplementary Table 1 — The primers for real-time PCR. The primers used for real-time PCR were listed. [file Image_1.tiff]
